# Examining Resilience in Those With and Without Suicidal Ideation

**DOI:** 10.3390/bs16020260

**Published:** 2026-02-10

**Authors:** Denny Meyer, Philip Sumner, Erica Neill, Andrea Phillipou, Wei Lin Toh, Tamsyn E. Van Rheenen, Susan L. Rossell

**Affiliations:** 1 Centre for Mental Health & Brain Sciences, Swinburne University of Technology, Melbourne, VIC 3122, Australia; psumner@swin.edu.au (P.S.); wtoh@swin.edu.au (W.L.T.); tamsyn.van@unimelb.edu.au (T.E.V.R.); srossell@swin.edu.au (S.L.R.); 2 Orygen, The National Centre of Excellence in Youth Mental Health, Melbourne, VIC 3052, Australia; erica.neill@orygen.org.au (E.N.); andrea.phillipou@orygen.org.au (A.P.); 3 Centre for Youth Mental Health, The University of Melbourne, Melbourne, VIC 3052, Australia; 4 Orygen Specialist Program, Parkville Youth Mental Health & Wellbeing Service, Melbourne, VIC 3052, Australia; 5 Department of Mental Health, St Vincent’s Hospital, Melbourne, VIC 3065, Australia; 6 Department of Mental Health, Austin Hospital, Melbourne, VIC 3084, Australia; 7 Department of Psychological Sciences, Swinburne University of Technology, Melbourne, VIC 3122, Australia; 8 Department of Psychiatry, Melbourne Medical School, University of Melbourne, Melbourne, VIC 3010, Australia; 9 InsideOut Institute, University of Sydney, Sydney, NSW 2006, Australia

**Keywords:** psychological resilience, transitional analysis, suicidal ideation, mental health

## Abstract

Self-report surveys were conducted in Australia between May 2020 and April 2024, allowing for an analysis of perceived psychological resilience in those with and without suicidal ideation (SI) during and after the COVID-19 pandemic. Linear mixed models were used to describe the factors associated with psychological resilience in these populations and in people experiencing transitions between SI states. Of the 1145 people who responded more than once to the survey, 879 (77%) always reported “never SI”, 84 (7%) always reported SI, while 182 (16%) reported SI for only some of their surveys. People who moved between SI states reported significantly lower psychological resilience than those who reported “never SI”, but significantly higher psychological resilience than those reporting SI in all their surveys. For participants always reporting SI, greater psychological resilience was significantly associated with greater hopefulness and quality of life, and less sleep than usual. In people who moved between SI states, greater psychological resilience was significantly associated with greater hopefulness, less psychological distress and lower likelihood of mental illness. Only participants with “never SI” reported better psychological resilience alongside consistent sleep and exercise quantities. These results have important implications for suicide prevention in Australia. However, bidirectional associations require further investigation.

## 1. Introduction

The concept of resilience relates to the ability of some people to recover and adapt better than others after experiencing a severely stressful event (Ahern et al., 2006). Psychological resilience is a component of the resilience construct that refers to an individual’s self-reported perceived ability to successfully cope with stressors and environmental changes, and to maintain psychological well-being in the face of adversity (Troy et al., 2023; Den Hartigh & Hill, 2022). In the research literature, psychological resilience is usually measured using self-report questionnaires. Psychological resilience during the COVID-19 pandemic has been the topic of several studies, which reported that it was related to modifiable lifestyle factors such as exercise, social support and sleep (e.g., Killgore et al., 2020; Park et al., 2021). Psychological resilience has been identified as a protective factor against increased alcohol use (Cusack et al., 2023) and drug addiction (Calpe-López et al., 2022).

Psychological resilience can extend to resilience within the context of suicide. Siegmann et al. (2017) found that positive mental health, satisfaction with life and perceived social support improved suicidal resilience in Chinese and German students. As reported by Han et al. (2022), mental and physical wellbeing also contribute positively to suicidal resilience. However, there are many other factors which have been associated with suicidality and suicidal ideation (SI), such as low hopefulness (Liu et al., 2014; Stark et al., 2022). In particular, it has been found that the relationship between COVID-19 stress and the presence of suicidal thoughts was much stronger for people with low psychological resilience and low hopefulness (Knowles et al., 2022). Lifestyle factors have also been linked to SI; alcohol consumption by Currier et al. (2016); and sleep disturbances by Bernert and Joiner (2007). Although Lee and Park (2025) found a negative relationship between SI and physical activity, in at least one study, no such relationship was found (e.g., Fabiano et al., 2023). During the COVID-19 pandemic, social support was a particularly important protective factor against SI (Selak et al., 2024; Zhang et al., 2022; Ayuso-Mateos et al., 2023).

However, SI is an inherently dynamic process (Bryan et al., 2019), especially for people with mental illness (Hallensleben et al., 2018). Liu et al. (2016) found no significant relationship between psychological resilience and SI in a longitudinal context, when controlling for depression, anxiety and rumination across two time points over a four-year period. Chen et al. (2023) found that although low psychological resilience could predict adolescent SI, SI could not predict psychological resilience over three surveys six months apart. Harris et al. (2021) also showed in a three-month study that, in people with a psychotic disorder but no mood disorder, the presence of SI was only evident when psychological resilience was low. Finally, Okechukwu et al. (2022) found that psychological resilience moderated the relationship between stress and SI, with significant positive associations between stress and SI at low and moderate levels of resilience, but no association when resilience was high. These findings suggest that the longitudinal examination of psychological resilience as it relates to SI is important.

The aim of this study was to examine this relationship in a set of surveys conducted in the general population of Australia between May 2020 and April 2024, a period beginning close to the onset of the COVID-19 pandemic in Australia. It was hypothesised that individuals who fluctuated between reporting and not reporting SI across surveys would report lower psychological resilience than those who never reported SI, but higher psychological resilience than those who always reported SI.

In addition, it was hypothesised that the factors contributing to psychological resilience would differ in four groups of individuals with different forms of SI presentation over time. As explained below, the identification of these four groups was data-driven, consisting of those who never reported SI, those who always reported SI and those who reported SI in only some of their surveys. This last group of participants with “mixed” SI reports was split according to whether or not the first survey completed reported SI. This allowed for a more nuanced monitoring of the initial impacts of the COVID-19 pandemic for participants with “mixed” SI reports. There is a paucity of work on whether predictors of SI differ in individuals who have fluctuating SI status during times of crisis. Therefore, this was explored in this Australian dataset with no explicit predictions.

## 2. Materials and Methods

### 2.1. Data Collection

This study utilised data from our COVID-19 and you: mentaL heaLth in AusTralia now survEy (COLLATE) project. The project comprised 18 surveys conducted between April 2020 and April 2024, on a monthly basis in the first year, every 4 months in the second year and annually thereafter. No data for SI were collected in the first survey (April 2020); so, that survey has been excluded here. The COLLATE project has been described in detail elsewhere (Tan et al., 2020), but, in brief, members of the Australian general public were invited to participate in anonymous online surveys, completed at their convenience and taking approximately 15–20 min. Online consent was obtained for the use of the personal and health information provided for the purposes explained. Each survey was active for 72 h from 9 am on the first day of the survey to 8:59 am on the fourth day of the survey (which was usually 1st–4th of the month unless falling on a public holiday). Only survey responses where the bulk of the survey was completed (typically about 95% of those where a serious attempt was made to complete the survey) were retained for data analysis. Only data directly related to the research questions in this study are presented here, and only participants who completed the survey on more than one occasion (N = 1145) were included.

In total, 879 participants consistently reported “never SI”, 84 consistently reported SI (“always SI”), and 182 reported fluctuations in SI across the surveys they completed. Participants with fluctuating SI were split into two groups with sample sizes of 88 and 94, respectively, for those with and without SI at the time of their first survey. These two groups were labelled “mixed (+/−)” and “mixed (−/+)”, respectively. It is these four groups of participants (SI groups) that were compared in this study, allowing for variation in the number of surveys completed for each participant. Psychological resilience was also compared for the “mixed (+/−)” and “mixed (−/+)” groups across their first “SI survey” and first “no SI survey” and by the ratio of positive to negative SI reports.

The COLLATE project received ethics approval from the Swinburne University Human Ethics Review Committee (approval number: 20202917-4107: 31 March 2020) and complied with the Declaration of Helsinki.

### 2.2. Measures

SI was measured using Yes/No responses to the question, “Have you experienced any suicidal thoughts in the last month?” As explained by Millner et al. (2015), the use of a single item to measure suicidal ideation is not ideal but is commonly done. Perceived psychological resilience was measured with the Brief Resilience Scale, with higher scores indicating greater psychological resilience. This is a six-item scale; each response was recorded from 1 = strongly agree to 5 = strongly disagree. This scale was developed by Smith et al. (2008) and had good reliability in the COLLATE project (α = 0.92) and many other studies (e.g., Smith et al., 2008 (α > 0.80); Chmitorz et al., 2018, (α = 0.85)).

Based on the above literature review of variables associated with psychological resilience and SI, the following demographic, lifestyle and psychological measures were included in this study.

#### 2.2.1. Demographic Measures

Gender was recorded as male, female or other. Participants were divided into three age groups at the time of their first survey completion: (i) a young age group between 18 and 24 years, (ii) a middle age group between 25 and 49 years, and (iii) an older age group of 50 years or older to accommodate the small percentage of participants over the age of 59.

A self-reported endorsement of mental illness, obtained in response to the question “Are you a person with a mental illness?”, was also considered in this study. There is evidence that individuals self-identifying as having bipolar disorder or depressive disorder typically meet criteria for each respective diagnosis (Kupfer et al., 2002; Sanchez-Villegas et al., 2008), suggesting that a self-reported measure for mental illness has validity. There was no attempt made to subdivide according to type of mental illness due to small numbers in some diagnoses, as indicated by Phillipou et al. (2020). A self-reported endorsement of physical illness, obtained in response to the question “Do you currently have a physical/medical health issue?”, was also considered in this study.

#### 2.2.2. Lifestyle Measures

Change in alcohol use was assessed with the following question: “In the past week, have there been any changes to the amount you are drinking?” For the analysis, a nominal variable was created to compare those who reported no change in their drinking to those who reported drinking less or more than usual in the past week.

Change in sleep quantity was measured using the question “In the past week have you experienced any significant changes in your sleeping patterns?” Again, in this study, we used a nominal variable to compare those who had experienced no change in their sleep patterns with those who were sleeping less or more than usual in the past week.

Change in the amount of physical activity was measured using the question “In the past week have you experienced any significant changes in your exercise behaviours?” Again, a nominal variable to compare those who had experienced no change in their exercise with those who had experienced an increase or decrease in their exercise in the past week was used.

Living situation included the following categories: living alone, living in a share house, couple with no children, couple with children, or single parent. However, for the analysis, a binary variable was created to compare those who reported living alone with those who lived with others. In addition, a variable for time in contact with other people “who do not live with you” was included as a binary “social support outside the home” variable, identifying those who spent at least two hours a day engaged in such contact.

Finally, current psychological support data were collected from the following question, “In the past four weeks, have you sought professional psychological support (e.g., from a GP, psychiatrist, psychologist, counsellor)?”, using responses that identified ongoing or newly initiated support, trying to establish support, or no support.

#### 2.2.3. Psychological Measures

Psychological distress was measured with the Depression Anxiety Stress Scales (DASS-21). This is a 21-item self-report measure developed by Lovibond and Lovibond (1995), with individual items scored on four-point Likert scales, 0 (did not apply to me at all in the last week) to 3 (applied to me very much or most of the time in the last week). The DASS-21 is a well-established measure with excellent reliabilities in the COLLATE study for the subscales involving depression (α = 0.933), anxiety (α = 0.873) and stress (α = 0.898).

Hopefulness was assessed using the Brief Positively Worded Hopefulness scale (Brief-H-Pos) developed by Everson et al. (1996), with “yes” responses counted as one and negative responses counted as zero. The scale contained two items and therefore had a range of 0 to 2, with higher scores indicating greater hopefulness.

Momentary affect, as measured by the Positive and Negative Affect Schedule, was also of interest (PANAS; Watson et al., 1988). The PANAS is a 20-item measure comprising 10 items measuring indicators of positive affect (PA) and 10 items measuring indicators of negative affect (NA). It is scored on the extent to which one has a particular feeling at that moment, with ratings on a 5-point scale ranging from very slightly or not at all (1) to extremely (5). A high score on the Positive Affect scale reflects an energetic and alert mood state, whereas a high score on the Negative Affect scale indicates greater levels of general distress. Items include “distressed” (NA scale) and “excited” (PA scale).

Finally, perceptions of quality of life (QoL) were considered as a proxy for life satisfaction as suggested by Malvaso and Kang (2022). QoL refers to the degree to which a person feels satisfied and comfortable with their life. It is a multidimensional concept that can be quantified through subjective assessment across four domains, namely, social, environmental, physical and psychological functioning (Skevington et al., 2004). QoL was measured using the EUROHIS-QOL 8-Item Index, a shortened version of the World Health Organisation Quality of Life Instrument (WHOQOL), with items relating to overall QoL, general health, energy, daily living activities, self-esteem, interpersonal relationships, finances, and home. Responses were measured on a 5-point Likert scale, from 1 = very dissatisfied to 5 = very satisfied, with higher scores indicating greater QoL. da Rocha et al. (2012) found that internal consistency for this measure ranged between 0.72 and 0.81 across six countries, but a higher value of 0.87 was found for the COLLATE project.

### 2.3. Statistical Analysis

Analyses were conducted using SPSS v30. A time series graph was used to describe how SI and psychological resilience varied over the 17 surveys included in this study and descriptive statistics were presented by SI group for the first survey completed by each participant, with crosstab and ANOVA tests used to test for significant SI group differences. Addressing the first hypothesis, the four SI groups were then compared in terms of psychological resilience across all completed surveys using a univariate linear mixed model analysis. The second hypothesis was addressed by first checking whether demographic, lifestyle and psychological measures were associated with psychological resilience as main effects or as moderators of the relationship between the SI groups and resilience, again using univariate linear mixed models. Several significant SI group moderation effects suggested that multivariate linear mixed models should be fitted for psychological resilience for each of the SI groups independently. In the first such model, only the demographic and lifestyle variables were included, with the psychological measures included only in the second such model.

## 3. Results

### 3.1. Descriptive Statistics

**Figure 1:**
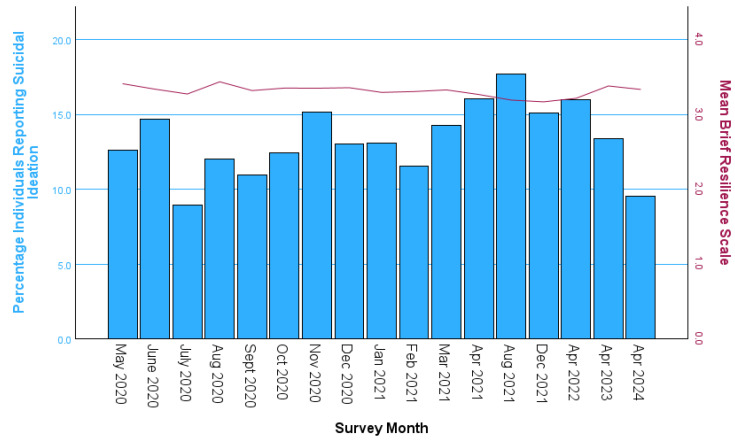
Variation in the percentage of individuals reporting suicidal ideation (SI) in blue and the mean psychological resilience score in red for each survey.

Figure 1 demonstrates the stable nature of the psychological resilience scale across all surveys and the more volatile nature of SI over this five-year period. On average, the number of survey responses included per month was 282, with a minimum of 179 in April 2024 and a maximum of 373 in February 2021.

Considering only the first survey completed by each participant in Table 1, the majority of the participants were female and middle-aged. Just over half the participants were located in Victoria at the time of their first survey, with 22.2% in New South Wales (NSW) and 27.5% located elsewhere in Australia. This is relevant because the capital of Victoria (Melbourne) bore the brunt for COVID-19 related lockdowns in Australia, as explained in Appendix A. Of the 1145 participants, 306 (26.7%) reported having one or more mental illness, 411 (35.9%) reported a physical health condition and 172 (15.0%) reported SI in the last week. For all these variables, there were significant differences between the SI groups with a large effect size in the case of mental illness (Cramer’s V = 0.444).

At their initial survey, the “always SI” group had the highest percentages for mental illness (75.0%) and for the 18–24 age group. The “mixed (+/−)” group had the highest percentage for other gender, the second highest percentage for age 18–24 years and for mental illness (60.2%). However, the “mixed (−/+)” group of participants was not far behind, with 50.0% reporting mental illness in their initial survey. The group with “never SI” had significantly fewer participants with a physical or mental health condition at the time of their initial survey.

In terms of the lifestyle variables at the time of the initial survey completed by each participant, there were significant differences between the SI groups for changes in sleep and exercise amounts in the last week, living situation and the seeking of professional psychological support in the last four weeks. However, the effect sizes (Cramer’s V) for these variables were not large. The “always SI” and mixed SI groups were significantly less likely to report no change in sleep quantity in the last week than the “never SI” group The “mixed (+/−)” group was significantly more likely to report less exercise in the last week than the “never SI” group and significantly less likely to report more exercise in the last week than the “never SI” group. The “never SI” group was significantly more likely to live with others than any of the other groups. Ongoing professional psychological support was most common for the “mixed (+/−)” and “always SI” groups. However, the percentages for participants who were without professional psychological support and not seeking it were also very high for these two groups (47.7% and 44.0%, respectively).

Considering only the first survey completed by each participant, significant differences were found between the SI groups for all the psychological measures in Table 2, with consistently middle values for the mixed SI groups. Effect sizes (*η^2^*) were particularly large for psychological distress and quality of life. There were no significant differences between the groups in terms of their first and last survey months; however, there were significant differences between the SI groups in terms of the number of surveys completed, with the highest number of survey completions for individuals in the “mixed (−/+)” group and the lowest number of survey completions for the “always SI” group.

A comparison between the “mixed (−/+)” and “mixed (+/−)” groups showed significantly higher psychological resilience for the “mixed (−/+)” group at the time of the first survey completed. The majority of the participants in both these groups (73%) had completed their first survey by the beginning of December 2020 and their survey with their first change in SI state by April 2021, with an average gap of 3.9 and 3.8 months between these surveys, respectively.

Importantly, the changes in psychological resilience observed between the first “SI survey” and first “no SI survey” were not significant for the “mixed (−/+)” group (*t*(93) = 0.892, *p* = 0.375)) or the “mixed (+/−)” group (*t*(87) = 0.822, *p* = 0.413). Also, as shown in Table 2, the levels of psychological resilience were persistently higher for the “mixed (−/+)” group than the “mixed (+/−)” group for their first surveys with and without SI. In addition, the percentage of surveys where SI was reported was significantly lower (*χ^2^*(1) = 8.62, *p* = 0.004) for the “mixed (−/+)” group than the “mixed (+/−)” group (37.0% versus 46.8%). Interestingly, a linear mixed model analysis showed that the ratio of positive to negative SI surveys was associated with significantly lower psychological resilience in the “mixed (−/+)” group (*t*(92) = 3.36, *p* < 0.001). However, there was no significant relationship between this ratio and psychological resilience in the “mixed (+/−)” group.

### 3.2. Comparison of SI Groups in Terms of Psychological Resilience over All Survey Completions

**Figure 2:**
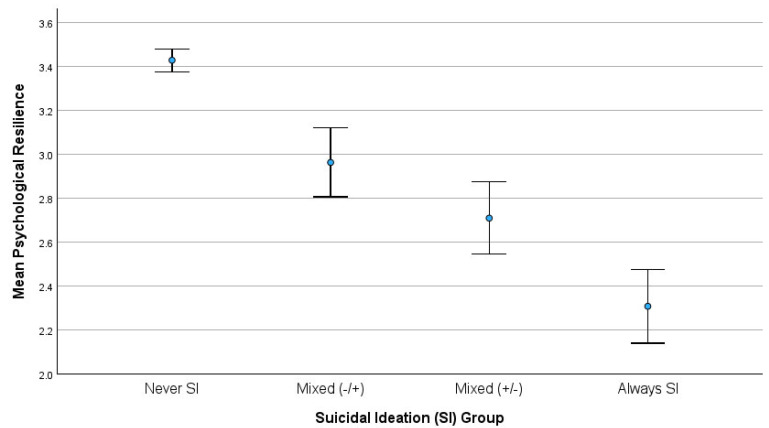
Relationship between mean psychological resilience and SI group with 95% confidence intervals.

As illustrated in Figure 2, significant differences in mean psychological resilience were observed for the four SI groups (*F*(3,1140) = 74.44, *p* < 0.001) when a linear mixed model was used to model this relationship over all survey completions. Significant differences were obtained for all pairwise comparisons of the four groups, providing support for the first hypothesis. There was considerable consistency in psychological resilience over time for individual participants, with 80% of the variation in this measure attributed to participant differences as opposed to individual changes between surveys (ICC = 0.80).

### 3.3. Moderation Tests for the Relationship Between Psychological Resilience and SI Group over All Survey Completions

In Table 3, univariate linear mixed models for psychological resilience were fitted with main and group SI moderation effects for each of the demographic, lifestyle and psychological measures individually. No significant main effects were found for gender, age or physical health condition when other variables were not controlled, but there were significant main effects for mental illness and for state. There were also significant main effects for changes in alcohol consumption in the last week and seeking professional psychological help in the last four weeks. In addition, all the psychological measures were found to have significant main effects.

Importantly, there were significant SI group moderation effects for mental illness, living situation, psychological distress and quality of life. These moderation results suggested that separate multivariate psychological resilience models were needed for each of the SI groups.

### 3.4. Multivariate Linear Mixed Models for Psychological Resilience for Each SI Group

Psychological resilience models were fitted separately for each of the SI groups using all the variables found in Table 1, Table 2 and Table 3 (except social support outside the home, which was not significant in any of the above tables). In the first model, only the demographic and lifestyle variables were included, with the results shown in Table 4.

In the “never SI” group, psychological resilience was significantly lower in females than in males and significantly higher for participants over the age of 50 years compared to younger participants aged 18–24 years. Psychological resilience was significantly lower for those with a mental illness and for those who slept more or less than usual in the last week. Also, significantly lower psychological resilience was reported by those who had exercised less than usual in the last week and those who were receiving ongoing professional psychological support.

For the mixed SI groups, psychological resilience was again significantly lower in those with a mental illness. For the “mixed (+/−)” group, an increase in alcohol consumption in the last week, living with others and ongoing psychological support were associated with lower psychological resilience. Finally, those who always reported SI showed no significant predictors of greater psychological resilience in Table 4, except reduced sleep in the last week and living in Victoria as opposed to New South Wales.

The multivariate linear mixed models with the psychological measures are shown in Table 5. These results suggest that for the “never SI” group, greater hopefulness, quality of life and positive affect were significantly associated with greater psychological resilience, while greater psychological distress and negative affect were significantly associated with reduced psychological resilience. For those in the “mixed (−/+)” and “mixed (+/−)” groups, greater hopefulness and less psychological distress were significantly associated with greater psychological resilience. Finally, for the “always SI” group, greater hopefulness and quality of life were significantly associated with greater psychological resilience.

## 4. Discussion

This study has compared four groups of survey participants, all of whom had completed the COLLATE survey at least twice over a five-year period beginning near the start of the COVID-19 pandemic. One of these groups never reported SI, while the remaining three groups reported SI in at least one of their surveys. As suggested by the literature (Stark et al., 2022; Knowles et al., 2022), psychological resilience was lower in the groups that reported SI, especially when the SI was sustained over all survey completions.

The results from the first survey completed by each participant also confirmed other previous findings. Hopefulness was significantly lower for the groups experiencing SI (Knowles et al., 2022; Liu et al., 2014; Stark et al., 2022), with disrupted sleep patterns (Bernert & Joiner, 2007), insufficient social support (Selak et al., 2024; Zhang et al., 2022; Ayuso-Mateos et al., 2023), and mental and physical illness (Han et al., 2022) also more likely in these groups. In support of the findings of Siegmann et al. (2017), it was found that, for the group that never reported SI, satisfaction with life (aka quality of life) was higher and psychological distress was lower.

In support of the findings of Lee and Park (2025), but contrary to those of Fabiano et al. (2023), reductions in exercise at the time of the first participant survey were more evident in the groups experiencing SI. Also, contrary to what was found by Currier et al. (2016), no significant differences for changes in alcohol consumption were found between the groups at the time of the first survey. Situational factors in some parts of Australia during the early surveys (that is, severe COVID-19 lockdown restrictions) may explain these contradictory findings. These lockdowns may also explain why the “never SI” group was more likely to have lived with others than any of the other groups, with social support outside the home not showing any significant differences between the four groups.

There were also significant differences in the levels of psychological resilience reported between the four groups of survey participants over all survey completions (n = 4792). Psychological resilience levels were reported as aligning with SI; that is, lowest with the group that always reported SI and highest with the group that never reported SI, and the two mixed SI groups fell between. These findings support the first hypothesis while also indicating that once SI is experienced, it must be expected that psychological resilience will be lower, even if SI is overcome in the future, as was the case for the “mixed (+/−)” group (Chen et al., 2023).

The majority of individuals in the “mixed” groups experienced a change in SI state well before the fourth Melbourne lockdown period (see Appendix A and Appendix B), suggesting that the differences between the “mixed (−/+)” and “mixed (+/−)” groups can perhaps be interpreted in the context of initial reactions to the COVID-19 pandemic. The “mixed (−/+)” group was able to initially avoid SI, perhaps due to their higher psychological resilience, but most of these participants (73%) still succumbed to SI within four months in the first year of the pandemic. In this group, more SI relapses, as measured using the ratio of positive to negative SI survey responses, were associated with lower psychological resilience. The “mixed (+/−)” group was unable to initially avoid SI, perhaps due to their lower psychological resilience, but the majority (coincidentally also 73%) managed to overcome their SI at least temporarily within four months in the first year of the pandemic.

Univariate Linear Mixed Model analyses were initially used to address the second hypothesis to determine what factors were associated with psychological resilience over this five-year period. Changes in exercise and sleep quantity, living situation and social support outside the home were not found to be individually associated with psychological resilience as suggested by some authors (Killgore et al., 2020; Park et al., 2021), perhaps due to the way in which these variables were defined, confounding with other variables or pandemic effects, as noted elsewhere. However, mental illness, state, changes in alcohol consumption, professional psychological support and all the psychological measures produced significant main effects. In addition, significant SI group moderation results were obtained for mental illness, living situation, psychological distress and quality of life, suggesting that separate multivariate models for psychological resilience should be fitted for each of the four SI groups.

In these models, the group reporting SI for all surveys was the only group for which mental illness was not significantly associated with reduced psychological resilience. Indeed, there was trending support for a positive relationship between mental illness and psychological resilience for this group. This can perhaps be attributed to the high percentage of participants with a mental illness in this group (75.0%). Participants in the “always SI” group without a mental illness had similar or even lower perceptions of their psychological resilience than those with a mental illness, perhaps because they had an undiagnosed mental illness. For the “always SI” group, reduced sleep, greater hopefulness and greater quality of life were associated with better psychological resilience, confirming the work of Ohayon et al. (2013), Berghöfer et al. (2020) and Huen et al. (2015). There was also a significant result for state, with participants resident in Victoria reporting higher psychological resilience than those in NSW. This was not expected because the lockdowns were more severe in Victoria than NSW. However, the sample size was very small for NSW (only nine participants); so, this result is perhaps not reliable.

The models for the two “mixed” groups, reporting transitions between SI states, both suggested lower psychological resilience in the case of mental illness, higher psychological distress and lower hopefulness. However, only for the group reporting SI in their initial survey (mixed +/−) was higher psychological resilience associated with living alone. The fear of COVID-19 infection if living with others at this time may have contributed to this result. Living with others might also have had a negative impact on psychological resilience if relationships at home were strained for any reason. This group also reported lower psychological resilience if they had been drinking more than usual in the previous week and if they were already receiving ongoing professional psychological support.

Finally, the model for those who had reported “never SI” indicated lower psychological resilience in those with mental illness and in those with changes in sleep quantity (more of less sleep than normal) and reduced exercise in the last week, together with ongoing professional psychological support. In addition, psychological resilience was found to be higher in more elderly people (50+) than in younger people (18–24), and higher in men than women. There have been numerous studies that support this age effect in general and during the pandemic in particular (e.g., Fuller & Huseth-Zosel, 2021). Karmann et al. (2023) attribute this greater resilience in older people to life experience providing better tools for overcoming adversity. Boardman et al. (2008) have attributed gender differences to higher levels of environmental mastery and self-esteem in men. Also, for this “never SI” group, it was found that psychological resilience was associated with all the psychological measures (hopefulness, psychological distress, quality of life, positive and negative affect), suggesting that it might be possible to build psychological resilience for this group by addressing all these issues in therapy, as well as through changes in sleep duration.

This study has clearly demonstrated that the factors associated with psychological resilience differed for the four SI groups, suggesting that a tailored approach, depending on the dynamic nature of SI presentation, could be helpful if psychological resilience building is to be attempted. However, the results suggest that hopefulness should be an important focus for all groups. For those reporting more entrenched SI and mental illness, quality of life needs to be an important focus. As explained by Berghöfer et al. (2020), aspects of health care that increase quality of life are essential for severely mentally ill patients, as complete freedom from their respective disorder(s) cannot be expected.

For people transitioning between SI states, reductions in psychological distress are important, suggesting the need for professional psychological support. The lack of engagement with professional psychological support by these two groups indicates that there is much scope to address this issue. At the time of the first survey, 60.6% of the “mixed (−/+)” group and 47.7% of the “mixed (+/−)” group were not yet even trying to find such support, let alone accessing such support.

Although this study has been successful in investigating the relationship between psychological resilience and SI, and in demonstrating what factors are important for improving psychological resilience for each of the SI groups, it does have some limitations. Firstly, the surveys were conducted over a long period of time, May 2020 to April 2024, at irregular time intervals, with major variations in the social environment caused by the COVID-19 pandemic. The lack of a significant correlation between social support outside the home and psychological resilience is thought to have been caused by the extreme lockdown conditions experienced in Australia (particularly Melbourne), amidst the other distortions caused by the pandemic. In particular, the rapid changes in SI state that were observed for the mixed SI groups, despite no significant changes in psychological resilience, may have been produced by the extreme stresses experienced during the pandemic. For at least 73% of these participants, changes in SI state were observed within an average period of less than four months.

The surveys included in this study were completed throughout this five-year period with variation in terms of the number of surveys completed by each participant. There were significant differences in the mean number of surveys completed by the four SI groups, although the effect size for these differences was very small (η^2^ = 0.023); so, this is unlikely to have impacted the results. More importantly, the sample sizes for the three groups reporting SI in at least one survey were relatively small, but sufficient to detect important differences between these groups.

Some of the measures used in this study might not be ideal. Millner et al. (2015) found that a single-item measure of suicide ideation does not meet standard definitions of suicide ideation, suggesting that an improved measure of suicidal ideation should be used in future studies. In addition, a quality-of-life measure was used as a proxy measure for life satisfaction. Future studies should endeavour to measure life satisfaction more directly, in addition to other variables linked to suicidal resilience, such as meaning in life, sense of responsibility and self-efficacy (Wang et al., 2022). In addition, only changes in the amount of drinking, sleep and exercise in the last week were considered in this study. This meant that the effects of continued problematic/beneficial lifestyle factors on psychological resilience could not be studied. It must also be acknowledged that changes in these lifestyle factors between surveys were not considered.

Finally, SI is a heterogeneous phenomenon. According to Rizk et al. (2018), those with brief SI are more stress-responsive. They may have greater emotional reactivity, and less cognitive control over their thoughts and emotions, and thus are at high risk for impulsive suicidal behaviour. On the other hand, those with chronic persistent SI are stress-non-responsive and tend to have more severe or chronic depression, but good cognitive control over their thoughts, allowing them to plan more carefully for their suicide attempts (Bernanke et al., 2017). The data for this study did not allow for a rigorous differentiation between those with brief and persistent SI. It is therefore not appropriate to assume that respondents in the “always SI” group had chronic persistent SI.

For the above reasons, it is suggested that further research is needed to establish to what extent the factors impacting psychological resilience differ in populations experiencing transient or persistent SI. Ideally, this research should also allow the direction of relationships between psychological resilience and SI to be more accurately assessed over time, as recommended by Chen et al. (2023). Lagged time series models and cointegration tests (Enders, 2004) are conventionally used to test for causality and directionality with economic data. More recently, network analysis has been used for investigating causal relationships in psychological studies (Hevey, 2018). Such methods may require the use of alternative data collection methods and technologies such as Ecological Momentary Assessment (EMA).

## 5. Conclusions

This five-year study has shown that there is a significant relationship between suicidal ideation and psychological resilience, with more entrenched suicidal ideation observed in those with lower psychological resilience. However, the factors significantly associated with psychological resilience appear to differ depending on the dynamic nature of the SI observed, prioritising different treatment foci for the building of psychological resilience in these SI groups. Clinicians should take into consideration any previous SI presentation when choosing the focus of the resilience therapy.

For those with more frequent SI, quality of life needs to be prioritised with attention paid to sleep and therapies that cultivate hope. For those with changes in SI states, treatments that increase hopefulness and decrease psychological distress need particular attention while addressing any mental illness concerns. For those experiencing SI as an initial reaction to an event such as the COVID-19 pandemic, increased drinking and living situation concerns should also be addressed. Finally, for those with no reports of SI, consistent quantities of sleep and exercise and better psychological health are associated with greater psychological resilience and should therefore be promoted. However, in view of the limitations of this study, more research is needed to confirm these results and to explore any bidirectional associations. For researchers investigating the relationships between SI and psychological resilience, the collection of data in a form that will allow for more sensitive testing for causality could be helpful.

## Figures and Tables

**Table 1:** Descriptive statistics (*n* (*%*)) for the demographic and lifestyle variables for the SI groups from initial survey completed by each participant.

	Categories	Never SI (*n* = 879)	Mixed (−/+) (*n* = 94)	Mixed (+/−) (*n* = 88)	Always SI (*n* = 84)	Total (*n* = 1145)	*χ* *^2^*	*df*	*p*-Value *	Cramer *V*
Gender	Male	276 (31.4)	22 (23.4)	26 (29.5)	21 (25.0)	345(30.1)	**24.65**	**6**	**<** **0** **.001**	**0** **.104**
	Female	596 (67.8)	69 (73.4)	56 (63.6)	60 (71.4)	781 (68.2)				
	Other	**7 (0.8)**	3 (3.2)	**6 (6.8)**	3 (3.6)	19 (1.7)				
Age	18–24	**113 (12.9)**	22 (23.4)	**26 (29.5)**	**25 (29.8)**	186 (16.3)	**40.55**	**6**	**<** **0** **.001**	**0** **.133**
	25–49	546 (62.3)	54 (57.4)	47 (53.4)	52 (61.9)	699 (61.2)				
	50+	**218 (24.9)**	18 (19.1)	15 (17.0)	**7 (8.3)**	258 (22.6)				
State	NSW	205 (23.5)	19 (20.2)	19 (21.9)	9 (11.0)	252 (22.2)	10.38	6	0.098	0.066
	VIC	438 (50.2)	50 (53.2)	42 (48.3)	41 (50.0)	571 (50.3)				
	Other	229 (26.3)	25 (26.6)	26 (29.9)	32 (39.0)	312 (27.5)				
Mental illness	No	736 (83.7)	47 (50.0)	35 (39.8)	21 (25.0)	839 (73.3)	**225.49**	**3**	**<** **0** **.001**	**0** **.444**
	Yes	**143 (16.3)**	**47 (50.0)**	**53 (60.2)**	**63 (75.0)**	306 (26.7)				
Physical illness	No	608 (69.2)	42 (44.7)	43 (48.9)	41 (48.8)	734 (64.1)	**42.64**	**3**	**<** **0** **.001**	**0** **.193**
	Yes	**271 (30.8)**	**52 (55.3)**	**45 (51.1)**	**43 (51.2)**	411 (35.9)				
Alcohol use in last week	No change	540 (61.9)	58 (61.7)	56 (65.1)	56 (67.5)	710 (62.5)	2.96	6	0.836	0.036
	Less	155 (17.8)	14 (14.9)	16 (18.6)	11 (13.3)	196 (17.3)				
	More	178 (20.4)	22 (23.4)	14 (16.3)	16 (19.3)	230 (20.2)				
Sleep quantity in last week	No change	**378 (43.1)**	**27 (28.7)**	**22 (25.0)**	**22 (26.2)**	449 (39.2)	**25.08**	**6**	**<** **0** **.001**	**0** **.105**
	Less	256 (29.2)	35 (37.2)	39 (44.3)	31 (36.9)	361 (31.6)				
	More	244 (27.8)	32 (34.0)	27 (30.7)	31 (36.9)	334 (29.2)				
Exercise amount in last week	No change	321 (36.6)	34 (36.2)	33 (37.5)	24 (28.6)	412 (36.0)	**14.92**	**6**	**0** **.019**	**0** **.081**
	Less	**240 (27.4)**	33 (35.1)	**36 (40.9)**	31 (36.9)	340 (29.7)				
	More	**316 (36.0)**	27 (28.7)	**19 (21.6)**	29 (34.5)	391 (34.2)				
Living situation	With others	**756 (86.0)**	**73 (79.3)**	**70 (80.5)**	**65 (77.4)**	964 (84.4)	**7.68**	**3**	**0.048**	**0.082**
	Alone	123 (14.0)	19 (20.7)	17 (19.5)	19 (22.6)	178 (15.6)				
Contacts outside home	>2 h/day	168 (19.2)	18 (19.1)	14 (15.9)	14 (16.7)	214 (18.7)	0.81	3	0.877	0.027
	≤2 h/day	727 (80.6)	78 (81.3)	75 (84.3)	73 (83.9)	953 (81.2)				
Professional psychological support in last 4 weeks	Ongoing	**125 (14.2)**	**24 (25.5)**	**29 (33.0)**	**36 (42.9)**	214 (18.7)	**113.41**	**9**	**<** **0** **.001**	**0** **.182**
	Newly initiated	**18 (2.1)**	**5 (5.3)**	**8 (9.1)**	4 (4.8)	35 (3.1)				
	Not yet	**21 (2.4)**	**8 (8.5)**	**9 (10.2)**	7 (8.3)	45 (3.9)				
	No	**714 (81.3)**	**57 (60.6)**	**42 (47.7)**	**37 (44.0)**	850 (74.3)				

* Chi-squared test of associations with Fisher–Freeman–Halton Exact *p*-value. **Significant results bolded.**

**Table 2:** Descriptive statistics for the psychological measures for the initial survey and survey completions for the SI groups.

	Mean (SD) for SI Groups					ANOVA Test			
	Never SI (*n* = 879)	Mixed (−/+) (*n* = 94)	Mixed (+/−) (*n* = 88)	Always SI (*n* = 84)	Total (*n* = 1145)	*F*(3,*df*)	*df*	*p*-Value	*η* *^2^*
**Psychological Measures**									
Psychological resilience	3.40 (0.84) ^a^	2.96 (0.85) ^b^	2.70 (0.80) ^c^	2.33 (0.79) ^d^	3.23 (0.89)	**61.50**	**1141**	**<0.001**	**0.139**
Hopefulness	1.62 (0.65) ^a^	1.28 (0.85) ^b^	0.93 (0.88) ^c^	0.79 (0.87) ^c^	1.47 (0.76)	39.11 *	**158**	**<0.001**	**0.140**
Psychological distress	22.86 (18.04) ^a^	41.28 (21.81) ^b^	57.32 (24.92) ^c^	67.73 (22.61) ^d^	30.32 (24.17)	220.6 *	**181**	**<0.001**	**0.290**
Positive affect	24 51 (8.19) ^a^	21. 40 (7.50) ^b^	20.16 (7.35) ^b^	16.42 (4.41) ^c^	23 33 (8.20)	65.73 *	**183**	**<0.001**	**0.090**
Negative affect	14.73 (5.48) ^a^	18.49 (7.59) ^b^	21.53 (9.52) ^c^	23.75 (9.80) ^c^	16.22 (7.08)	45.23 *	**159**	**<0.001**	**0.152**
Quality of life	29.94 (5.46) ^a^	25.04 (6.02) ^b^	22.57 (5.83) ^c^	20.36 (5.01) ^d^	28.27 (6.35)	**127.09**	**1141**	**<0.001**	**0.250**
**Survey Completion** **Variables**									
1st survey completed ^#^	7.21 (4.46) ^ab^	6.67 (4.22) ^a^	6.90 (4.27) ^ab^	8.26 (4.48) ^b^	7.22 (4.44)	2.19	1141	0.088	0.006
Last survey completed ^#^	13.22 (4.38) ^ab^	14.45 (3.72) ^b^	13.47 (4.01) ^ab^	12.90 (4.54) ^a^	13.32 (4.32)	2.63	1141	0.049	0.004
Number surveys completed	4.12 (3.07) ^b^	5.53 (3.32) ^c^	4.26 (2.92) ^b^	3.31 (2.09) ^a^	4.19 (3.05)	11.26 *	**176**	**<0.001**	**0.024**
1st “SI survey” ^#^	NA	10.57 (4.54) ^a^	6.90 (4.27) ^b^	NA	NA	**31.59 ^@^**	**180**	**<0.001**	**0.145**
1st “no SI survey” ^#^	NA	6.67 (4.22) ^a^	10.66 (4.61) ^b^	NA	NA	**37.08 ^@^**	**180**	**<0.001**	**0.166**
Resilience for 1st “SI Survey”	NA	2.90 (0.89)	2.70 (0.80)	NA	NA	2.64 ^@^	180	0.106	0.014
Resilience for 1st “no SI Survey”	NA	2.96 (0.85)	2.76 (0.88)	NA	NA	2.46 ^@^	180	0.118	0.013

* ANOVA test with SQRT transformation, a robust Welch test used because an assumption of homogeneity of variance rejected. ^a,b,c,d^ Student–Neuman–Keuls pairwise comparisons; ^#^ surveys numbered consecutively from 2 to 18 for May 2000 to April 2024. ^@^ Comparison of “mixed (−/+)” and “mixed (+/−)” groups using *F*(1,*df*); **significant group comparisons bolded**; NA = not applicable.

**Table 3:** Main effects and potential moderators of the relationship between psychological resilience and SI group over all survey completions.

	Main Effect				Moderation Effect by SI Group			
Potential Predictors of Resilience	*F*-Value	Numerator *df*	Denominator *df*	*p*-Value	*F*-Value	Numerator *df*	Denominator *df*	*p*-Value
**Demographics**								
Gender	1.59	2	2385	0.203	0.44	6	2559	0.850
Age	0.25	2	1892	0.780	0.85	6	2180	0.534
Mental illness	**14.96**	**1**	**4768**	**<** **0** **.001**	**3.82**	**3**	**4766**	**0** **.010**
Physical illness	2.21	1	4475	0.137	0.14	3	4475	0.935
State	**3.79**	**2**	**1895**	**0** **.023**	1.97	3	1970	0.067
**Lifestyle Variables**								
Alcohol change in last week	**4.41**	**2**	**4216**	**0** **.012**	1.03	6	4118	0.406
Sleep change in last week	2.51	2	4002	0.081	1.67	6	3971	0.124
Exercise change in last week	1.306	2	4034	0.273	2.04	6	3980	0.057
Living situation	0.10	1	4225	0.748	**2.81**	**3**	**4244**	**0** **.038**
Social support outside home	0.28	1	4048	0.594	0.55	3	4026	0.650
Professional psych support in last 4 weeks	**4.54**	**3**	**4261**	**0** **.003**	1.43	9	4159	0.168
**Psychological Measures**								
Hopefulness *	**94.82**	**1**	**4364**	**<** **0** **.001**	1.03	3	4261	0.377
Psychological Distress *	**117.48**	**1**	**4733**	**<** **0** **.001**	**2.61**	**3**	**4512**	**0** **.050**
Quality of Life	**140.75**	**1**	**4724**	**<** **0** **.001**	**3.40**	**3**	**4699**	**0** **.017**
Positive Affect *	**54.37**	**1**	**4394**	**<** **0** **.001**	1.48	3	4346	0.217
Negative Affect *	**103.67**	**1**	**4644**	**<** **0** **.001**	1.81	3	4510	0.143

* SQRT transformation; **significant results bolded**.

**Table 4:** Linear mixed model analysis for psychological resilience by SI group including only the demographic and lifestyle variables.

	Comparison	Never SI (*n* = 879)		Mixed (−/+) (*n* = 94)		Mixed (+/−) (*n* = 88)		Always SI (*n* = 84)	
		Estimated Difference	Std Error	Estimated Difference	Std Error	Estimated Difference	Std Error	Estimated Difference	Std Error
**Demographic** **Variables**									
Gender	Female vs. Male	−0.114 *	0.055	−0.044	0.194	−0.089	0.179	−0.157	0.169
	Other vs. Male	0.009	0.127	0.004	0.268	−0.051	0.299	−0.352	0.248
Age	25–49 vs. 18–24	0.087	0.062	0.043	0.127	0.069	0.155	−0.129	0.172
	50+ vs. 18–24	0.164 *	0.074	0.040	0.185	−0.201	0.235	−0.304	0.345
Mental Illness	Yes vs. No	−0.212 ***	0.038	−0.244 *	0.083	−0.240 *	0.112	0.207 #	0.124
Physical Illness	Yes vs. No	−0.034	0.023	−0.048	0.067	−0.033	0.078	−0.046	0.094
State	VIC vs. NSW	0.045	0.057	0.262	0.175	−0.089	0.170	0.573 *	0.263
	VIC vs. Other	0.036	0.057	0.315 #	0.181	0.308 #	0.176	0.091	0.190
**Lifestyle Variables**									
Drinking in last week	Less vs. No Change	−0.010	0.022	0.046	0.070	−0.083	0.087	−0.072	0.111
	More vs. No Change	−0.016	0.023	−0.086	0.071	−0.212 *	0.095	−0.143	0.105
Sleep quantity in last week	Less vs. No change	−0.051 **	0.019	−0.078	0.059	−0.025	0.071	0.196 *	0.089
	More vs. No change	−0.071 ***	0.020	−0.067	0.062	−0.078	0.078	0.060	0.088
Exercise amount in last week	Less vs. No change	−0.042 *	0.019	0.053	0.059	0.045	0.073	0.137	0.090
	More vs. No change	−0.020	0.019	−0.051	0.059	0.032	0.074	0.130	0.091
Living situation	Live with others vs. Live Alone	−0.022	0.040	0.204	0.139	−0.255 *	0.112	0.067	0.166
Prof psych support in last 4 weeks?	Ongoing vs. No	−0.070 *	0.031	0.043	0.077	−0.179 *	0.087	−0.095	0.116
	Newly initiated vs. No	−0.043	0.046	−0.073	0.102	−0.144	0.123	−0.132	0.143
	Not yet but trying vs. No	−0.006	0.049	0.179 #	0.104	0.142	0.130	−0.043	0.129

Adjusted *p*-values: # *p* < 0.10, * *p* < 0.05, ** *p* < 0.01, *** *p* < 0.001; **significant results bolded**.

**Table 5:** Linear mixed model analysis for psychological resilience by SI group including only the psychological measures.

	Never SI (*n* = 879)		Mixed (−/+) (*n* = 94)		Mixed (+/−) (*n* = 88)		Always SI (*n* = 84)	
**Psychological Measures**	**Coefficient**	**Std Error**	**Coefficient**	**Std Error**	**Coefficient**	**Std Error**	**Coefficient**	**Std Error**
Hopefulness (SQRT)	0.063 **	0.021	0.133 **	0.049	0.125 *	0.061	0.163 *	0.068
Psychological Distress (SQRT)	−0.042 ***	0.007	−0.066 **	0.023	−0.041 *	0.021	−0.031	0.037
Quality of Life	0.023 ***	0.003	0.007	0.007	0.009	0.008	0.019 *	0.009
Positive Affect (SQRT)	0.090 ***	0.014	−0.002	0.044	0.030	0.049	0.092	0.066
Negative Affect (SQRT)	−0.074 ***	0.019	**−0.072 #**	0.041	−0.006	0.048	**−0.092 #**	0.053

# *p* < 0.10, * *p* < 0.05, ** *p* < 0.01, *** *p* < 0.001; **significant and trending results bolded**.

## Data Availability

The original contributions presented in this study are included in this article. Further inquiries can be directed to the corresponding author.

## Abbreviations

The following abbreviations are used in this manuscript:

COLLATE	COVID-19 and you: mentaL heaLth in AusTralia now survEy
PA	Positive Affect
NA	Negative Affect
QoL	Quality of Life
“mixed (−/+)”	A mix of SI states with “no SI” for first survey completed
“mixed (+/−)”	A mix of SI states with “SI” for first survey completed
SQRT	Square root transformation to reduce skewness in distribution
NSW	New South Wales
VIC	Victoria
SI	Suicidal Ideation

## Appendix A

### Appendix A.1. COVID-19 Lockdown Statistics for Melbourne, Victoria, Australia

Melbourne had six lockdowns, totalling 262 days: (Boaz, 2021)

- Lockdown 1: 26 March to 12 May 2020—43 days;
- Lockdown 2: 8 July to 27 October 2020—111 days;
- Lockdown 3: 12 February to 17 February 2021—5 days;
- Lockdown 4: 27 May to 10 June 2021—14 days;
- Lockdown 5: 15 July to 27 July 2021—12 days;
- Lockdown 6: 5 August to 21 October 2021—77 days (Al Jazeera, 2021; Kelly, 2021);
- Lockdowns were phased out after 70% of the population was vaccinated in October 2021 with most public health restrictions removed after vaccinating 90% of its population in December 2021.

### Appendix A.2. COVID-19 Lockdown Statistics for NSW, Australia

NSW had two lockdowns, totalling 183 days:

- Lockdown 1: March 18 to July 1 2020—76 days;
- Lockdown 2: June 23 to October 11 2021—107 days.

By 18 March 2020, Australia had recorded a cumulative total of 567 COVID-19 cases and 5 deaths, and the federal government declared the virus to be a biosecurity emergency. (COVID-19 Data, 2020; Our World in Data, 2020; Parliament of Australia, 2020).

All of Australia’s state/territory government subsequently implemented lockdowns.

## Appendix B

### Appendix B.1. Government Response to the COVID-19 Pandemic

A detailed description in terms of the pandemic period, oral antiviral treatments, COVID-19 tests, vaccination, reporting, national coordination, expert advice, policies and procedures, public health information and support can be found in: https://www.health.gov.au/topics/covid-19/about/what-we-did?language=en (accessed on 1 February 2026).

### Appendix B.2. Australia’s Response to COVID-19

Stodart and Duckett (2022) describe the interventions which were implemented to contain the transmission of the disease, detailing the successes and failures.

### Appendix B.3. A Brief Overview of Australia’s Economic Response to COVID-19

The Australian government provided economic support for employers and employees, households and individuals during the pandemic. This report describes some of the key economic measures that were provided by the Australian Federal Government. However, there were other economic support measures provided by the state governments, such as the restaurant vouchers provided by the Victorian State Government. https://www.hlb.global/a-brief-overview-of-australias-economic-response-to-covid-19/ (accessed on 1 February 2026).

### Appendix B.4. The COVID-10 Pandemic: 2020 to 2021

This report by the Reserve Bank of Australia describes the economic effects of the pandemic. Economic activity was severely impacted, unemployment increased but inflation declined. Policy responses involved changes in monetary and fiscal policy. However, the economy rebounded rapidly as the virus was contained, restrictions were eased and the vaccines were rolled out, supported by government transfers and tax incentives for investment. The recovery in Victoria and New South Wales was more restrained due to the longer lockdowns and greater impacts of international border closures. Supply chain disruptions, stronger inflation and some labour shortages were experienced in the wake of the pandemic and there were some longer-lasting effects affecting where people work and study and online commerce, reducing the demand for commercial office and retail space. https://www.rba.gov.au/education/resources/explainers/pdf/the-covid-19-pandemic-2020-to-2021.pdf (accessed 1 February 2026).
